# A Chinese herbs complex ameliorates gut microbiota dysbiosis induced by intermittent cold exposure in female rats

**DOI:** 10.3389/fmicb.2022.1065780

**Published:** 2022-11-30

**Authors:** Lu Jin, Xiangyu Bian, Weiyun Dong, Renren Yang, Che Jing, Xi Li, Danfeng Yang, Changjiang Guo, Weina Gao

**Affiliations:** Department of Nutrition and Food Hygiene, Institute of Environmental and Operational Medicine, Tianjin, China

**Keywords:** Chinese herbs complex, cold exposure, gut microbiota, intestinal barrier function, HPO axis

## Abstract

Cold is a common source of stress in the alpine areas of northern China. It affects the microbial community, resulting in the invasion of pathogenic microorganisms and intestinal diseases. In recent years, studies have reported that Chinese herbal extracts and their fermentation broth have a significant beneficial effect on gut microbiota. This study aimed to investigate the probiotic effect of a self-designed Chinese herbs complex on the gut microbiota of rats exposed to cold. The rats were treated with intermittent cold exposure and Chinese herbs complex for 14 days, and the gut microbiota composition and other parameters were assayed. The 16s ribosomal DNA high-throughput sequencing and analysis confirmed that the Chinese herbs complex positively improved the gut microbiota. We found that cold exposure could lead to significant changes in the composition of gut microbiota, and affect the intestinal barrier and other physiological functions. The relative abundance of some probiotics in the genus such as *Roseburia, Parasutterella, and Elusimicrobium* in rats treated with Chinese herbs complex was significantly increased. Serum D-lactic acid (D-LA) and lipopolysaccharide (LPS) were increased in the cold exposure group and decreased in the Chinese herbs complex-treated group. Moreover, the Chinese herbs complex significantly increased the protein expression of occludin. In conclusion, the Chinese herbs complex is effective in restoring the gut microbiota caused by cold exposure, improving the function of the intestinal barrier, and may act as a prebiotic in combatting gut dysbiosis.

## Introduction

Stress is a state caused by harmful stimuli from internal or external environments and leads to a disturbed internal environment (Selye, 1936). As one of the common stressors, the cold exposure response can be diverse depending on gender differences. Especially to women, a short-term cold exposure study showed that some metabolic and hormonal (such as plasma glucose, leptin, and adiponectin) were changed more pronounced in women compared to men (Ježová et al., 1996). Cold exposure can cause menstrual disorders and dysmenorrhea, as well as reproductive hormone disorders and a variety of gynecological diseases. Cold exposure also affects the microbial community (Chevalier et al., 2015; Ziętak et al., 2016), resulting in the invasion of pathogenic microorganisms and intestinal diseases. Chevalier et al. demonstrated that fecal microbial composition was changed in response to a cold environment (6°C) in rats (Chevalier et al., 2015). In piglets, cold exposure-induced microecological imbalance and intestinal damage caused diarrhea and other diseases and even led to death (Zivkovic et al., 2011; Gresse et al., 2017). Zhang et al. (2011) found that chickens raised under low temperatures exhibited edema, hyperemia, and epithelial damage in the intestinal mucosa. Some studies have found that some traditional Chinese herbs, such as hawthorn, can regulate gut microbiota (Nazhand et al., 2020). However, to date, few studies have investigated the beneficial effects of Chinese herbs complexes on microecological imbalance and intestinal damage.

The hypothalamus-pituitary-ovary axis (HPO axis) is a major regulatory pathway in female mammals. Under the regulation of the HPO axis, there are regular changes in the female physiological cycle. The hypothalamic-pituitary-thyroid axis (HPT axis) is critical for maintaining homeostasis under various stresses. Gut microbiota affects neurotransmitters like noradrenaline, which can reduce serum thyroid-stimulating hormone (TSH) levels (Wostmann, 2020), and play an important role in the female reproductive endocrine system by interacting with estrogen. Therefore, we hypothesized that gut microbiota may interact with the HPO and HPT axis under cold exposure, and the Chinese herbs complex can regulate the secretion of HPO and HPT axis-related hormones in female rats by restoring the changes of gut microbiota caused by intermittent cold exposure.

The compatibility between monomers of traditional Chinese medicine enhances the efficacy of drugs, expand the scope of treatment, and adapt to complex conditions. For example, *Angelica* is an herb used in traditional Chinese medicine to enrich blood, promote blood circulation (Wang et al., 2013). Safflower, the tubular flower of *Carthamus tinctorius*, has been shown to promote blood circulation, remove blood stasis (Li et al., 2020). The combination of the two enhance the power of promoting blood circulation, regulating blood, and has the effect of regulating HPO axis hormone (Lixia et al., 2019). Semen persicae has a positive role in regulating blood flow and relaxing the bowels (Jiang et al., 2017). Safflower cooperated with semen persicae, was widely used to treat and modulate blood deficiency patterns such as menstrual disorders, and modulate the immune system. Hawthorn break congestion and remove abdominal fullness (Tang et al., 2016). When combined with semen persicae, it can complement each other and has the function of regulating the HPO axis hormone (Liu, 2005).

The purpose of this study was to reveal the effects of a Chinese herbs complex on the gut microbiota and the intestinal barrier in female rats under cold exposure. Furthermore, the relationship among Chinese herbs complex, gut microbiota, and hormone axis were also explored.

## Materials and methods

### Chinese herbs complex

Chinese herbs complex was formulated based on the principles specified by the Pharmacopeia of the People’s Republic of China (2015). The extracts of safflower, peach kernel, *Angelica*, and hawthorn were purchased from Xi’an Huilin Bio-tech Co., Ltd. (Xi’an, China). The dosage of each extract was calculated according to the Pharmacopeia of the People’s Republic of China as indicated in Table 1. The solution prepared was stored at 4°C until use.

**TABLE 1:** Composition of the Chinese herbs complex.

Extract	Dosage
Safflower	16.67 mg/kg
Peach kernel	16.67 mg/kg
*Angelica*	16.67 mg/kg
Hawthorn	25 mg/kg
Ferrous lactate	0.33 mg/kg
Folic acid	6.67 μg/kg
Vitamin B_12_	0.2 μg/kg

### Animals and treatments

**FIGURE 1:**
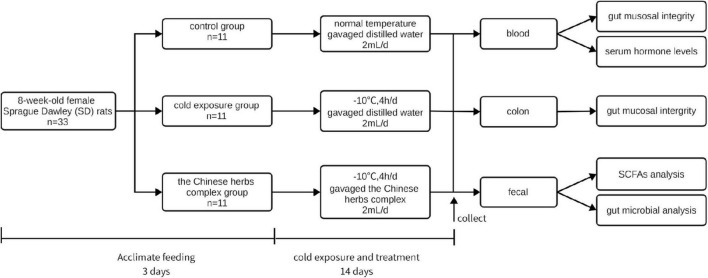
Chart of the experimental protocol. Thirty-three 8-week-old female Sprague Dawley (SD) rats (body weight 185.82 ± 5.89 g) were housed at a temperature of 23°C ± 1°C and relative humidity of 40% to 60% with a 12-h light/dark cycle. The animals had free access to tap water and diet for 5 days before being randomly assigned to the control group (C), cold exposure group (CE), and Chinese herbs complex group (CH) based on body weight. The rats in the C and CE groups were intragastrically administrated distilled water, and the rats in the CH group were given Chinese herbs complex by gavage once a day. After the treatment, the cold exposure group and Chinese herbs complex group were exposed to −10°C in a cabin for 4 h every day, and the treatment time lasted for 14 days.

This study was approved by the ethics committee of the Tianjin Institute of Environmental and Operational Medicine. All procedures were performed by the current Chinese legislation on the care and use of laboratory animals. Thirty-three 8-week-old female Sprague Dawley (SD) rats (body weight 185.82 ± 5.89 g) were obtained from the Beijing Vital River Laboratory Animal Technology Co., Ltd. (Beijing, China). The rats were housed at a temperature of 23°C ± 1°C and relative humidity of 40 to 60% with a 12-h light/dark cycle. The rats were acclimatized for 5 days and randomly assigned to the control group (C), cold exposure group (CE), and Chinese herbs complex group (CH) based on body weight. The rats in the C and CE groups were intragastrically administrated distilled water, and those in the CH group were given Chinese herbs complex solution by gavage once a day. After the treatment, the cold exposure group and Chinese herbs complex group were exposed to −10°C in a cabin for 4 h every day lasts 14 days. During the experimental period, body weight and dietary intake were recorded routinely. At the end of the experiment, all rats were sacrificed under ether anesthesia. Blood samples were collected immediately from the retrobulbar venous plexus. Serum samples were obtained by centrifugation and stored at −80°C. Fecal samples were collected from the rectum under sterile conditions, snap-frozen in liquid nitrogen, and stored at −80°C until analysis. The colonic tissue was fixed in 4% paraformaldehyde fix solution (PFA) at 4°C (Figure 1).

### Analysis of serum hormone levels

Sandwich enzyme-linked immunosorbent assay kits (Shanghai Jianglai Biotechnology Co., Ltd., Shanghai, China) were used to determine the levels of serum thyroid stimulating hormone (TSH), triiodothyronine (T3), thyroxine (T4), luteinizing hormone (LH), follicle-stimulating hormone (FSH), estradiol (E2), progesterone (Prog), D-lactic acid (D-LA) and lipopolysaccharide (LPS) by strictly following the manufacturer’s instructions. The absorbance at 450 nm wavelength was measured using a microplate reader (BioTeck, USA).

### Histological examination and immunohistochemistry of colonic mucosa

Hematoxylin and eosin (HE) staining was performed according to previously (Shi et al., 2020). A light microscope was used to capture images of the tissue sections [Leica DM4 B (Leica, Germany)]. The height of the villi was recorded by ImagePro Plus 5.1 software.

Immunohistochemistry was performed using the procedure described previously by Qu et al. (2021) More than three visual fields were randomly selected for observation under the microscope. Quantitative analysis of the target proteins was conducted using Image J software (version 1.5.7, National Institutes of Health, USA).

### Quantification of colonic short-chain fatty acids

Short-chain fatty acids (SCFAs), including acetate, propionate, butyrate, and isovalerate, were measured using a gas chromatography method reported previously by Zhao and Nyman (2006) and (Zhao et al., 2018). Briefly, 0.1 g colonic content was diluted with 1 mL deionized water, followed by vortexing and centrifugation at 10,000 rpm for 10 min. The supernatant was analyzed using a gas chromatographer (Agilent Technology 7890A) on a chromatographic capillary column (60 m × 0.25 mm × 0.25 μm) under the following conditions: initial temperature of 100°C for 0.5 min, followed by heating to 180°C at 10°C/min for 1 min, and heating for 240°C at 20°C/min for 5 min. The signal was detected at 270°C using a flame ionization detector. Hydrogen, air, and nitrogen as makeup gases were 40 ml/min, 300 ml/min, and 25 ml/min, respectively. Two-methyl butyraldehyde was used as the internal standard.

### Western blotting

Total protein extracts from colon tissues were prepared using ice-cold lysis buffer. The extracted proteins were denatured by boiling at 95°C for 5 min in a sample loading buffer. Equal amounts of proteins (30 μg) were loaded into the wells of sodium dodecyl-polyacrylamide gel electrophoresis gel for separation by electrophoresis and then transferred to a polyvinylidene fluoride membrane. After blocking with 5% skim milk in TBST buffer for 2 h at room temperature, the blots were probed with primary antibodies overnight, followed by incubation with the corresponding secondary antibody for 1 h. The blotting signals were detected using Pierce™ ECL Western Blotting Substrate (Thermo Fisher Scientific, Waltham, MA, USA) and imaged using Amersham Imager 680 (Amersham Pharmacia Biotech, Inc., Piscataway, NJ, USA). Image Pro Plus 6.0 software was used to analyze the blots, and the results were expressed as the ratio of the optical density of the target protein to that of GAPDH. The antibodies used in this experiment were listed in Table 2.

**TABLE 2:** The antibodies used in Western blotting.

Antibody	Company	Number	Fold dilution
Anti-occludin antibody	Biogot technology, co., Ltd. (Nanjing, China)	Catalog no. ap6005	1:1000
Anti-E-cadherin antibody	Biogot technology, co., Ltd. (Nanjing, China)	Catalog no. Bs1098	1:1000
Anti-MUC2 antibody	Abcam Ltd. (Cambridge, UK)	Catalog no. Ab272692	1:1000
Anti-GAPDH antibody	Cell Signaling Technology Ltd. (Boston, MA, USA)	Catalog no. 4970	1:1000
Antibody-anti-rabbit immunoglobulin G and horseradish peroxidase-linked antibody	Cell Signaling Technology Ltd. (Boston, MA, USA)	Catalog no. 7074	1:3000

MUC2, mucin 2.

### Analysis of gut microbiota

Five colon feces from each group were used for the analysis of the intestinal microbiota.

The microbial community was analyzed using prokaryotic 16S ribosomal DNA gene (16s rDNA) sequencing. Genomic DNA was extracted from the colonic contents using the TIANamp Soil DNA Kit (Tiangen Biotech, China, catalog no. DP336), and the total DNA was purified using a DNA purification Kit (Tiangen DNA gel extraction kit, China, catalog no. DP209). The concentration of the genomic DNA was measured using the Qubit dsDNA HS Assay Kit. The genomic DNA obtained was stored at −20°C until further processing. Approximately 30−50 ng of DNA was used to generate amplicons using a MetaVx Library Preparation kit (GENEWIZ, Inc., Suzhou, China). The V3 and V4 hypervariable regions of the 16s rDNA were selected to generate amplicons and taxonomy analysis was performed using the following primers: forward, 5′-CCTACGGRRBGCASCAGKVRVGAAT -3′, and reverse, 5′-GGACTACNVGGGTWTCTAATCC-3′. The indexed adapters were added to the end of the 16S rDNA amplicons to generate an indexed library, ready for downstream next-generation sequencing on the Illumina MiSeq platform. An Agilent 2100 Bioanalyzer (Agilent Technologies, Palo Alto, USA) was used to determine the library, and a Qubit 2.0 Fluorometer (Invitrogen, Carlsbad, USA) was used to measure the library concentration. Sequencing was performed using a 2 × 300 paired-end configuration on an Illumina MiSeq instrument (Illumina, San Diego, CA, USA). The MiSeq Control Software embedded in the MiSeq instrument was used for image analysis and base calling.

The QIIME data analysis package was used for the 16s rDNA data analysis. Sequences were grouped into operational taxonomic units using the clustering program VSEARCH (1.9.6) against the Silva 119 database clustered at 97% sequence identity. The Ribosomal Database Program classifier was used to assign a taxonomic category to all operational taxonomic units at a confidence threshold of 0.8. Alpha and beta diversity analyses were conducted using the QIIME software (version 1.7.0) and R software (version 2.15.3), and principal coordinate analysis was performed using unweighted UniFrac. The abundance at the phylum, class, and genus levels were calculated, and changes in composition from baseline were expressed as Log2 (n-folds change). Linear discriminant analysis of the effect size (LEfSe) was used to analyze the differences in microbial community structure among the groups. Furthermore, the Spearman correlation coefficient test performed correlation analysis between the relative abundance of microbiota taxa at the genus level and biochemical parameters.

### Statistical analysis

Statistical differences were analyzed using a one-way analysis of variance (ANOVA) of variance followed by the least-significant difference (LSD) *post-hoc* test or the Dunn-Bonferroni *post-hoc* method to further compare the difference among groups. Data are presented as the means ± standard error of the mean (SEM). SPSS 22.0 software (SPSS Inc., Chicago, IL, USA) was used. Statistical significance was considered at *p* < 0.05.

## Results

### Cold exposure mildly increased the food intake and decreased the body weight of rats

**FIGURE 2:**
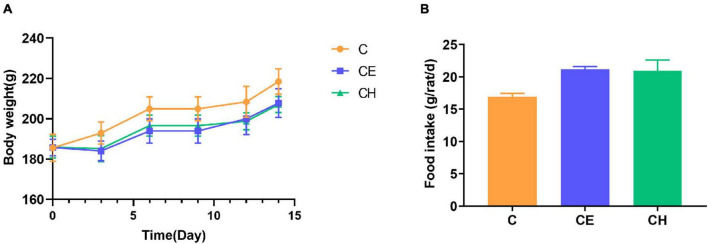
Changes in body weight and food intake. **(A)** Body weight; **(B)** food intake; Data are represented as the mean ± standard deviation (*n* = 8); C, control group; CE, cold exposure group; CH, Chinese herbs complex group.

During the experiment, the body weight of the CE and CH groups increased slower than the control group, though no statistical difference was found (Figure 2A). Although there was no significant difference among the three groups, the food intake of the cold exposure groups was mildly more than the control group at the end of the trial (Figure 2B).

### Chinese herbs complex alleviates the levels of serum repression hormones induced by cold exposure

**FIGURE 3:**
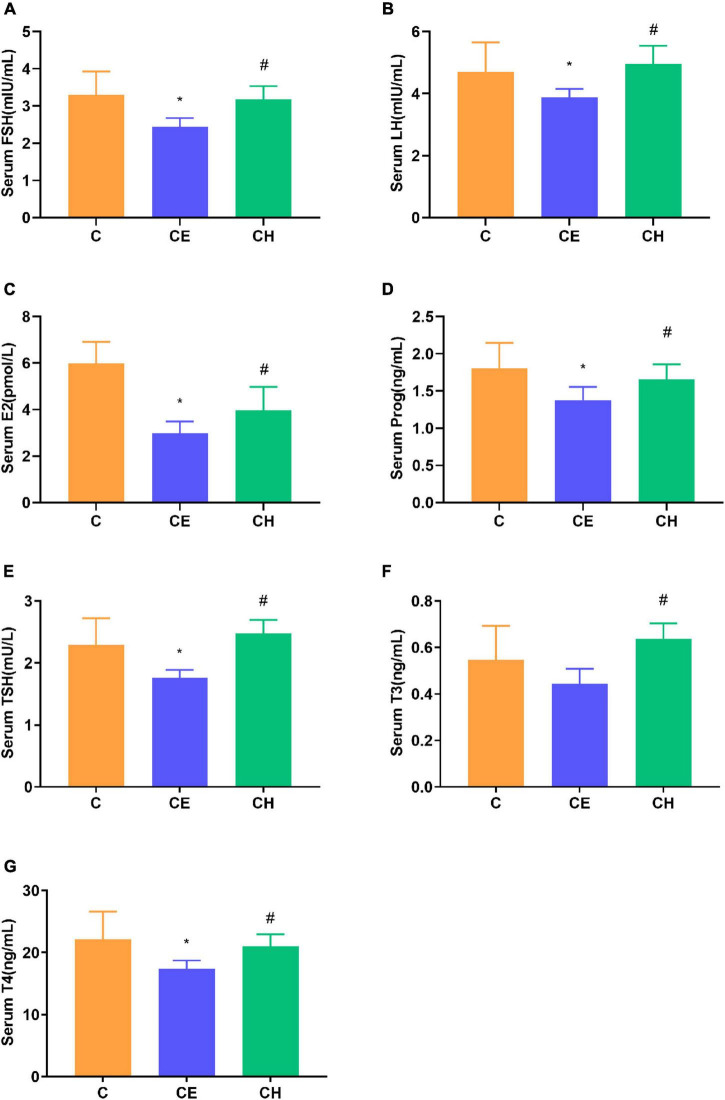
Alterations of the serum hormone levels. **(A)** FSH, follicle-stimulating hormone; **(B)** LH, luteinizing hormone; **(C)** E_2_, estradiol; **(D)** Prog, progesterone; **(E)** TSH, thyroid stimulating hormone; **(F)** T3, triiodothyronine; **(G)** T4, thyroxine. Data are represented as the mean ± standard deviation (*n* = 8). *Means are significantly different vs. C group (*p < 0.05); ^#^Means are significantly different vs. CE group (^#^*p* < 0.05); C, control group; CE, cold exposure group; CH, Chinese herbs complex group.

To investigate whether the cold exposure and Chinese herbs complex-treatment might influence the serum levels of hormones on the HPO and HPT axis. In this study, serum LH, FSH, E2, Prog, TSH, and T4 levels decreased significantly in the CE group compared with the C group. In the CH group, the serum contents of LH, FSH, E2, Prog, TSH, T3, and T4 increased significantly compared with the CE group (Figure 3). These results demonstrate that Chinese herbs complex promote the secretion of hormones related to the HPO and HPT axis which inhibited by cold exposure.

### Chinese herbs complex improves histological morphology of the colonic mucosa in rats exposed to cold exposure

**FIGURE 4:**
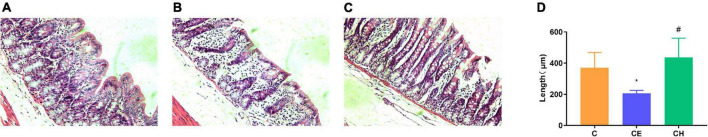
Changes in histological morphology of the colonic mucosa at the end of the experiment. Histological morphology of the colon (HE, ×200). **(A)** C group; **(B)** CE group; **(C)** CH group; **(D)** Comparison of the villus length of colonic mucosa among different groups; Data are presented as the mean ± standard deviation (*n* = 5). The means with different superscript letters are significantly different based on one-way ANOVA with LSD *post-hoc* analysis; *Means are significantly different vs. C group (*p < 0.05); ^#^Means are significantly different vs. CE group (^#^*p* < 0.05); C, control group; CE, cold exposure group; CH, Chinese herbs complex group.

Observation of the colonic mucosa under a light microscope revealed that the rat treated with cold exposure displayed severe epithelial injury, including distortion of the crypts and loss of goblet cells (Figures 4A–C). The numbers of villi and glands were also remarkably reduced in the CE group compared with the C group. The Chinese herbs complex improved mucosal morphology and increased the length of the villi (Figure 4D).

### Chinese herbs complex prevented loss of intestinal mucosa integrity in rats exposed to cold exposure

**FIGURE 5:**
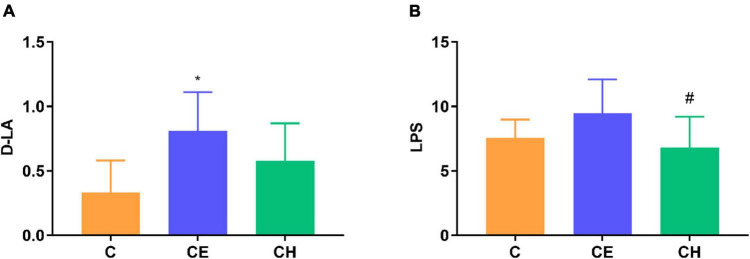
Serum D-lactic acid (D-LA) and lipopolysaccharide (LPS) levels; **(A)** Serum D-LA level; **(B)** Serum LPS level; *Means are significantly different vs. C group (*p < 0.05); ^#^Means are significantly different vs. CE group (^#^*p* < 0.05); Data are represented as the mean ± standard deviation (*n* = 8); C, control group; CE, cold exposure group; CH, Chinese herbs complex group.

To assess whether cold exposure affects the epithelial barrier function, we examined the epithelial permeability of the intestine in rats. Serum D-LA, a marker of intestinal mucosal integrity, is suggestive of changes in gastrointestinal permeability. LPS levels was measured to characterize systemic inflammation. We observed that serum D-LA levels in the CE group were higher than in the C group (*p* < 0.05). Compared with the CE group, serum LPS levels declined significantly in the CH group (*p* < 0.05) (Figure 5).

**FIGURE 6:**
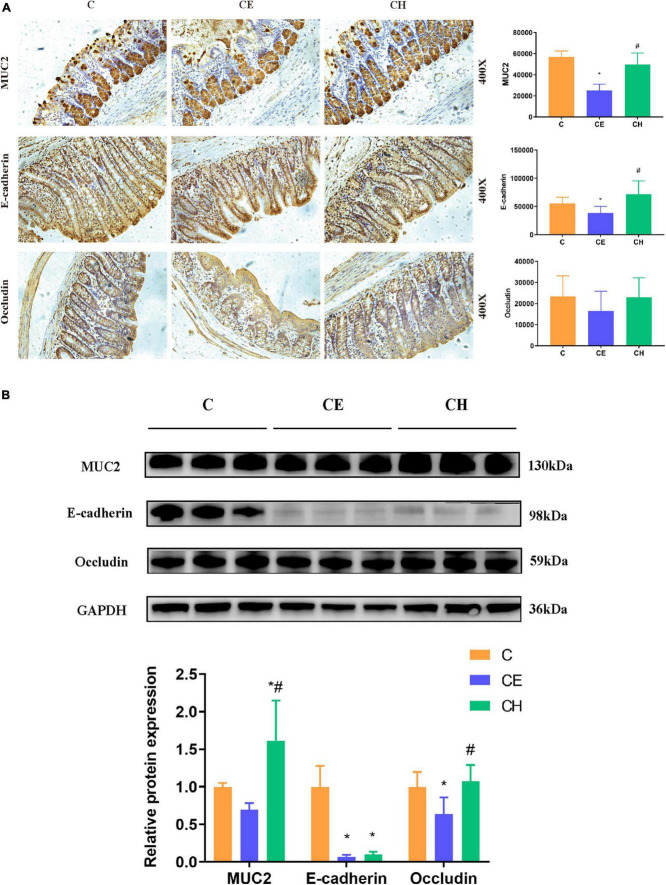
Protein expression of MUC2, E-cadherin, and occludin in the colon of rats treated with Chinese herbs complex for 14 days. **(A)** Representative images of immunohistochemical staining of MUC2, E-cadherin, and Occludin in colon samples from different experimental groups (scale bar, 250 mm). Data are presented as the mean ± standard deviation (*n* = 5), analyzed using one-way ANOVA with LSD *post-hoc* analysis. **(B)** Western blotting for E-cadherin, MUC2, occludin, and GAPDH; relative quantification of E-cadherin, MUC2 and occludin. Data are presented as the mean ± standard deviation (*n* = 3). The means with different superscript letters are significantly different based on one-way ANOVA with Dunn-Bonferroni *post-hoc* analysis; *Means are significantly different vs. C group (*p < 0.05); ^#^Means are significantly different vs. CE group (^#^*p* < 0.05); C, control group; CE, cold exposure group; CH, Chinese herbs complex group.

The colonic epithelium expresses mainly mucin 2 (MUC2) in large amounts which is the most important factor determining the goblet cell morphology (Hasnain et al., 2010). Immunohistochemistry results showed that colonic proteins expression of E-cadherin and MUC2 were notably decreased in the CE group in comparison to the control group (*p* < 0.05). The two proteins were up-regulated remarkably by the Chinese herbs complex (*p* < 0.05) (Figure 6A). The protein expression of E-cadherin, occludin and MUC2 were evaluated by Western blotting, results showed a similar trend toward the immunohistochemistry results (Figure 6B).

### Chinese herbs complex affects fecal short-chain fatty acid levels

In the CE group, the concentrations of acetic acid, propionic acid, isobutyric acid, and isovaleric acid did not change significantly after cold exposure. However, butyric was significantly increased in the CH group compared to the C group (Table 3).

**TABLE 3:** Short-chain fatty acids (SCFAs) levels in the colonic content.

Conditions	Acetic acid	Propionic acid	Isobutyric acid	Butyric acid	Isovaleric acid
C	1303.65 ± 256.89	217.06 ± 70.50	13.30 ± 1.17	299.30 ± 133.63	10.95 ± 1.80
CE	1254.73 ± 215.05	226.35 ± 79.96	14.29 ± 1.10	536.89 ± 122.83*	13.57 ± 1.37
CH	1443.27 ± 141.06	234.93 ± 72.87	18.66 ± 1.51	609.68 ± 118.74*	15.31 ± 1.67

*Means are significantly different vs. C group (*p < 0.05); C, control group; CE, cold exposure group; CH, Chinese herbs complex group.

### Chinese herbs complex improves gut microbiota dysbiosis caused by cold exposure

**FIGURE 7:**
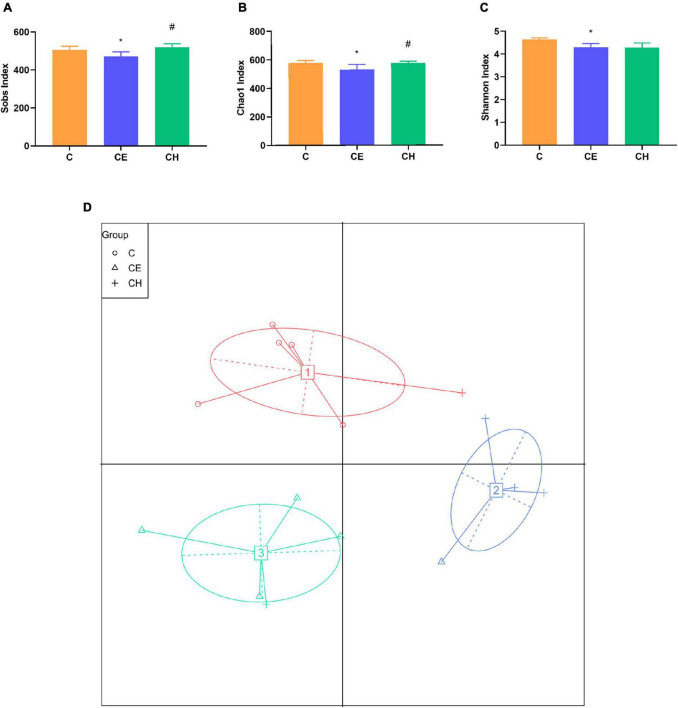
The diversity of gut microbiota in rats (*n* = 5); **(A–C)** diversity indices of microbial communities in fecal samples, box plots showed differences in the microbiome diversity among C, CE, and CH groups in terms of the sobs index, Chao1 index and Shannon indexes; **(D)** the effects of intermittent cold exposure on the fecal microbial communities; *Means are significantly different vs. C group (*p < 0.05); ^#^Means are significantly different vs. CE group (^#^*p* < 0.05); C, control group; CE, cold exposure group; CH, Chinese herbs complex group.

Our results demonstrated the beneficial effects of Chinese herbs complex on the gut microbiome after cold exposure. Sobs, Shannon, and Chao1 indexes were decreased significantly in the CE group compared to the C group (Figures 7A–C). However, Chinese herbs complex treatment significantly increased the Sobs and Chao1 indexes compared to the CE group. There was no statistically significant difference in the indexes of Sobs, Chao1 and Shannon between the C and CH groups. These data suggest that cold exposure significantly decreases the richness and evenness of gut microbiota and the Chinese herbs complex is effective in restoring the intestinal microbiota dysbiosis caused by cold exposure.

The principal coordinate analysis plot is used to display the separation among different groups. The results showed a clear separation of the community structure of fecal microbiota among the three groups, in which both the C and CH groups were well separated from the CE group (Figure 7), demonstrating that the composition of gut microbiota varied greatly among different groups.

### Chinese herbs complex modulates the composition of gut microbiota

**FIGURE 8:**
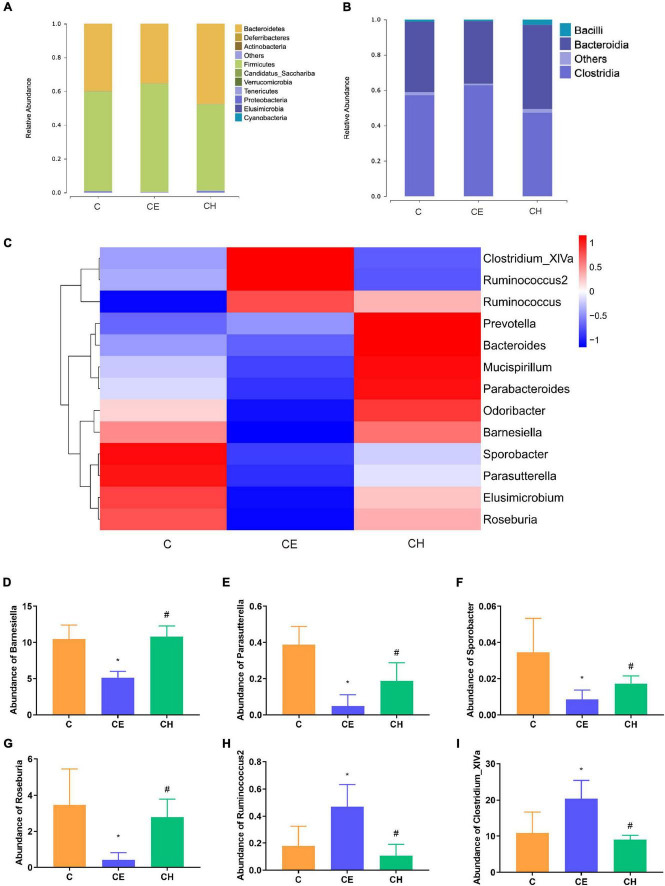
Relative abundance levels of the gut microbial community. **(A)** Phylum level; **(B)** class level; **(C)** genus level (*n* = 5); **(D)** abundance of *Barnesiella*; **(E)** abundance of *Parasutterella*; **(F)** abundance of *Sporobacter*; **(G)** abundance of *Roseburia*; **(H)** abundance of *Ruminococcus2*; **(I)** abundance of *Clostridium_XlVa*; *Means are significantly different vs. C group (*p < 0.05); ^#^Means are significantly different vs. CE group (^#^*p* < 0.05); C, control group; CE, cold exposure group; CH, Chinese herbs complex group.

At the phylum level, 10 bacterial phyla were identified. The CE group presented significantly lower levels of Bacteroidetes and Proteobacteria as well as a higher level of Firmicutes (*p* < 0.05) compared to the C group. These changes were partially reversed by the Chinese herbs complex (Figure 8A).

At the class level, the relative abundance of *Bacteroidia* and *Bacilli* were decreased, and *Clostridia* was significantly increased in the CE group in comparison with the C group (*p* < 0.05). In the CH group, these changes were recovered to some extent (Figure 8B).

At the genus level, the relative abundance of *Elusimicrobium*, *Mucispirillum*, *Odoribacter*, and *Parabacteroides* was deprived in the CE group, compared to the C group. And they were restored in the CH group (Figure 8C). The relative abundance of *Barnesiella*, *Parasutterella*, *Sporobacter*, and *Roseburia* were decreased, and *Ruminococcus2* and *Clostridium_XlVa* were increased in the CE group (*p* < 0.05), in comparison with the C group (Figures 8D–I).

**FIGURE 9:**
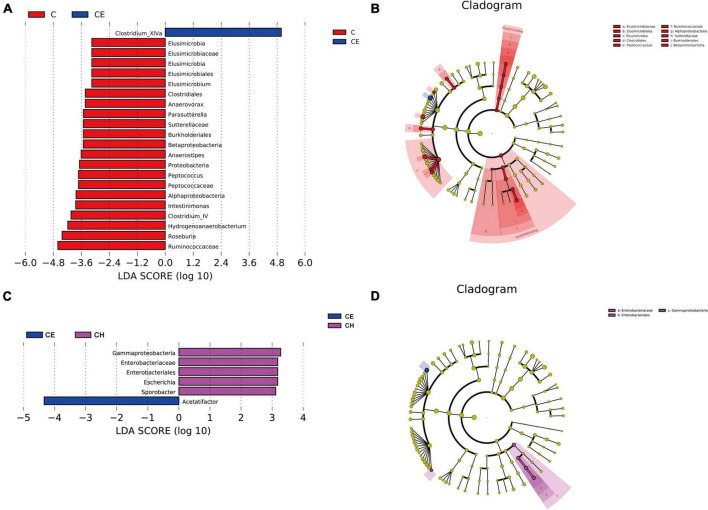
Linear discriminant analysis of the effect size (LEfSe) analysis of the gut microbiota among three groups. **(A,B)** The taxonomic cladogram was obtained from the LEfSe analysis of gut microbiota in different groups; **(C,D)** The LDA effect size of more than four was used as a threshold for the LEfSe analysis. C, control group; CE, cold exposure group; CH, Chinese herbs complex group.

Next, LEfSe was performed to further identify biomarkers and characterize the difference in the gut microbiome among the three groups. *Clostridium_XlVa* and *Ruminococcaceae* were higher in the C and CE groups, respectively (Figure 8A). Compared with the CE group, *Gammaproteobacteria* was dominant in the CH group (Figure 8C). According to the LEfSe analysis, these abundant taxa can be considered potential biomarkers for the corresponding groups (linear discriminant analysis (LDA) score > 4.0; *p* < 0.05) (Figure 9).

### Correlation between the gut bacteria and serum hormone levels and intestinal barrier parameters

**FIGURE 10:**
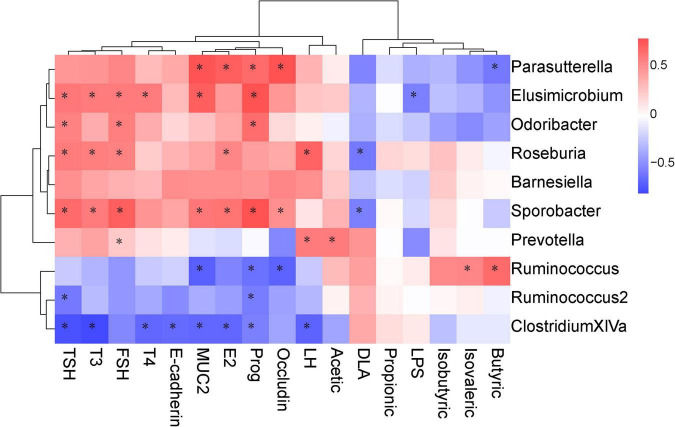
Correlations between the biochemical indicators and the operational taxonomic units (OTUs). The intensity of the color indicates the degree of correlation between the OTU abundance and the biochemical indicator as evaluated by Spearman’s correlation. Significant correlations are indicated with an asterisk (*) in the squares. The OTU taxonomy is listed on the right.

Spearman correlation analysis was performed to identify the possible correlation between specific bacterial taxa and biochemical parameters, such as SCFAs, intestinal mucosal integrity markers, serum hormone level and tight junction proteins. The results showed that there was a positive correlation between *Ruminococcus* abundance and butyric, isovaleric levels. Prevotella was positively correlated with acetic acid. In terms of intestinal epithelial integrity, Elusimicrobium was negatively correlated with serum LPS and positively correlated with MUC2 protein expression. *Sporobacter* was negatively correlated with serum D-LA and positively correlated with occludin protein expression. In addition, Parasutterella was positively correlated with occludin and MUC2 protein expression. In terms of serum hormones, Elusimicrobium, Roseburia, and *Sporobacter* were positively correlated with serum TSH, T3, and FSH (Figure 10).

## Discussion

In this study, 14-day intermittent cold exposure disrupted intestinal mucosal integrity, caused an increase in intestinal permeability, disordered the gut flora. It also caused hormonal disorders of the HPO axis and HPT axis in female rats, which was consistent with the results of previous studies (Zhang et al., 2011; Gresse et al., 2017; Xu et al., 2018; Wang et al., 2020). The Chinese herbs complex was able to reverse the observed negative effects by improved the imbalance of gut flora and increase the relative abundance of beneficial bacteria, such as *Roseburia* and *Sporobacter*. In addition, a significant enhancement was observed in the integrity of colonic mucosa in rats. These findings demonstrates that the Chinese herbs complex is beneficial to gut microbiota and intestinal barrier in female rats exposed to cold exposure.

The Shannon and Chao1 index were applied to assess the diversity and abundance of the microbial communities in each group, whereas high microbial diversity represents a robust and stable ecosystem (Lozupone et al., 2012) and low alpha diversity is associated with pathological states (Iszatt et al., 2019; Lee H. C. et al., 2019). Sobs, Shannon, and Chao1 index were decreased significantly following exposure to cold. This observation showed that cold exposure decreased microbial diversity. Similar results were found in mice and piglets (Zivkovic et al., 2011; Chevalier et al., 2015; Gresse et al., 2017). After treatment with the Chinese herbs complex, the Chao1 and Sobs index of the CH group were increased to a significant degree. These results demonstrates that the Chinese herbs complex enhances the diversity and evenness of the intestinal microbiota.

The composition of intestinal flora was significantly changed after cold exposure. LEfSe analysis indicated that the *Clostridium XIVa* and *Acetatifactor* were the biomarkers of the cold exposure group. *Clostridium XIVa* was reported to be associated with colitis and allergic diarrhea in mice (Atarashi et al., 2013). Transplanting fecal microbiota rich in *Acetatifactor* into healthy wildtype mice led to colonic inflammation (Lee C. et al., 2019; Yusufu et al., 2021). Chinese herbs complex declined the relative abundance of *Clostridium XIVa* and *Acetatifactor*, suggesting that the Chinese herbs complex inhibits the growth of harmful bacteria. Previous studies have shown that *Barnesiella* and *Parasutterella* played a potentially beneficial role in the mucosal secretion (Ju et al., 2019). Similarly, *Prevotella*, *Barnesiella*, and *Roseburia* plays a beneficial role in the intestinal environment by promoting the production of SCFAs, especially butyric acid (Sakamoto et al., 2009; Shi et al., 2017). *Elusimicrobium* is considered as probiotic, which improves the imbalance of intestinal flora and restore intestinal barrier function (Shi et al., 2017). Previous study showed that administration of *Roseburia* restored gut barrier dysfunction through upregulating Occludin and MUC2, which prevented the translocation of LPS (Seo et al., 2020). Intestinal colonization with *Barnesiella* confers resistance to intestinal domination and bloodstream infection with vancomycin-resistant *Enterococcus* (VRE) (Ubeda et al., 2013; Kawakami et al., 2020). After cold exposure, the relative abundance of *Barnesiella*, *Parasutterella*, and *Roseburia* was significantly decreased. Notably, the relative abundance of *Elusimicrobium* was deprived. LEfSe analysis also indicated that *Roseburia* was one of the biomarkers in the control group, and its relative abundance was significantly increased in the Chinese herbs complex group. The Chinese herbs complex could significantly increase the relative abundance of the flora mentioned above, suggesting that it is affecting in promoting the health of the intestinal environment by increasing the abundance of beneficial bacteria and decreasing the abundance of harmful bacteria in the colon.

Butyric acid participates in the regulation of colon homeostasis and helps to produce mucin and antimicrobial peptides as well as increase the expression of tight-junction proteins in the epithelial barrier (Vanhoutvin et al., 2009), and strengthen intercellular tight junctions together with thyroid hormones (Fröhlich and Wahl, 2019). Since *Roseburia*, *Prevotella* and *Barnesiella* are also important butyrate-producing bacteria (Zhang et al., 2020; Bae et al., 2022), and are remarkably enriched after the Chinese herbs complex treatment. These results suggests that the Chinese herbs complex improves intestinal homeostasis by regulating the abundance of SCFAs-producing bacteria in the intestine.

Gut epithelial tight junction protein and mucous secretion are both important for gut barrier function, isolating pathogens from gut epithelial cells and preventing infections and inflammations (Sicard et al., 2017). MUC2 is the predominant secretory mucin produced by colon goblet cells, which contributes to barrier integrity (Einerhand et al., 2002). Occludin and MUC2 proteins are recognized as markers of epithelial and mucous barrier functions (Sicard et al., 2017). Serum D-LA is a marker of intestinal mucosal integrity. LPS levels was measured to characterize systemic inflammation. These two markers indirectly manifested the changes of gastrointestinal permeate. In the present study, rats exposed to cold displayed epithelial injury, including distortion of the crypts and loss of goblet cells which are closely related to the function of the intestinal barrier. These results indicated that cold exposure damaged the intestinal structure and affected the integrity of the intestinal epithelium. The results were concordant with Pääkkönen and Leppäluoto (2002). Our results showed that the level of serum D-LA and LPS was significantly increased and the protein expression of MUC2 and Occludin in the colon was significantly decreased in the CE group. In the Chinese herbs complex-treated rats, the serum level of D-LA and LPS was decreased, the protein expression of MUC2 and Occludin was increased, the morphology of colonic mucosa was improved and the length of villi was increased significantly. Spearman correlation analysis showed that the protein expression of MUC2 and Occludin were significantly correlated with *Parasutterella* and *Sporobacter*, and MUC2 was also significantly correlated with *Elusimicrobium* and *Barnesiella*. Members of the genus *Sporobacter* and *Barnesiella* are described as strict anaerobes and are known to degrade aromatic compounds and produce short-chain fatty acids and remove harmful bacteria from the intestines (Grech-Mora et al., 1996). *Elusimicrobium* was thought to be beneficial bacteria, whose increase can protect the intestinal barrier (Shi et al., 2017). A study showed that *Parasutterella* may exert potential beneficial effects on intestinal mucosal homeostasis by elevating the level of hypoxanthine (Ju et al., 2019). These results demonstrate that cold exposure damages rat integrity of the intestinal epithelium through downregulating proteins involved in tight junction and mucus generation. The Chinese herbs complex has a beneficial effect in improving the integrity of the intestinal epithelium by favoring beneficial bacteria such as *Barnesiella*, *Parasutterella*, and *Elusimicrobium*.

A healthy gut microbiota not only has beneficial effects on the integrity of intestinal epithelium but also on thyroid function. Thyroid function is regulated by the hypothalamus-pituitary-thyroid axis, and the abnormal secretion of thyroid hormones causes a range of pathological and physiological changes (Li et al., 2010). Thyroid hormones, such as T3 play an important role in growth, and considered to be one of the important regulators for the development and differentiation of epithelial cells of the intestinal mucosa (Meng et al., 2001; Daher et al., 2009; Li et al., 2010). Studies have found that thyroid disorders often coexist with the disruption of intestinal epithelial barrier (Lerner et al., 2017; Qin et al., 2017), and dysbiosis of the microbial composition (Ishaq et al., 2017; Zhao et al., 2018). The imbalance of gut flora is also closely related to the abnormal secretion of thyroid hormones, which mainly inhibits the secretion of TSH and thus affects the secretion of HPT-axis-related hormones (Cao et al., 2014; Ishaq et al., 2017; Zhao et al., 2018). Previous studies demonstrated that *Elusimicrobium* and *Roseburia* acted in maintaining the integrity of the gut epithelium and the intestinal barrier by promoting the secretion of mucus and the production of butyric acid (Grech-Mora et al., 1996; Shi et al., 2017; Fröhlich and Wahl, 2019). The levels of serum TSH and T4 decreased significantly after cold exposure, indicating that cold exposure affected the secretion of hormones in the HPT axis. The levels of serum TSH, T3, and T4 were significantly increased after the Chinese herbs complex treatment. Spearman correlation analysis also showed that the levels of serum TSH and T3 were positively correlated with *Elusimicrobium*, *Roseburia*, and *Sporobacter*. These data indicate that Chinese herbs complex increases the abundance of related beneficial bacteria, which in turn improves the integrity of the intestinal epithelium and influence the production of hormones such as TSH and T3.

The gut microbiota plays an important role in the reproductive endocrine system by interacting with estrogens. Female reproductive function is regulated by the hypothalamus-pituitary-ovary (HPO) axis. During stress, the female reproductive system can be malfunctioned (Kinsey-Jones et al., 2009; Yang et al., 2017). Antibiotics decrease the secretion of estrogen (Adlercreutz et al., 1984). Estrogen or estrogen-like compounds decrease LPS by the gut microbiome and gut permeability, and modify gut epithelial barrier integrity in mice (Homma et al., 2005; Kaliannan et al., 2018). Several experiments suggested that *Bacteroides fragilis* had a positive correlation with serum LH (Strandwitz et al., 2019; Liang et al., 2021). *Bacteroides fragilis* increased gamma-aminobutyric acid, in which acts on the receptors of GnRH neurons in the hypothalamus to stimulate the secretion of LH (Strandwitz et al., 2019; Liang et al., 2021). Cold exposure leads to the levels of serum LH, FSH, E2, and Prog decreased significantly, indicating that cold exposure affected the secretion of HPO axis hormones. However, the hormones related to the HPO axis in rats treated with the Chinese herbs complex were significantly increased. The results of Spearman correlation analysis showed that the levels of serum LH, FSH, and E2 were positively correlated with *Roseburia* and *Sporobacter*, suggesting that the prescription of the Chinese herbs complex promote the secretion of hormones related to the HPO axis by increasing the abundance of related probiotics.

It has been reported that Chinese herbal extracts and their fermentation products broth have a significant prebiotic effect on gut flora (Liu et al., 2013; Eom et al., 2014; Gong et al., 2020). *Angelica*, semen persicae, and hawthorn significantly improve gut microbiota and increase the number of beneficial bacteria (Guowen et al., 2003; Li et al., 2016; Xu et al., 2017; Chen, 2020; Duan et al., 2020). Ferulic acid and amygdalin are the main active components of *Angelica sinensis* and peach kernel, respectively. They can promote the production of butyric acid by increasing the abundance of *Proteobacteria* (Liu et al., 2013; Liye et al., 2019; Xinhao et al., 2022), significantly ameliorated colonic inflammation, and restructured the gut microbiome and microbial metabolism (Opyd et al., 2017; Tian et al., 2022). Concurrently, the butyrate levels increased in this treatment. In contrast, the increase in both short-chain fatty acids and probiotics favored intestinal epithelial integrity. This is probably due to the combined effects of both substances. We shall further investigate the effect of these single component.

In conclusion, the present study indicates that cold exposure dysregulates intestinal barrier function by disturbing the balance of intestinal flora and impairing the secretion of hormones in the HPO and HPT axis. The Chinese herbs complex improve the dysbiosis of intestinal microflora caused by cold exposure and enhance the integrity of intestinal mucosa. This study reveals that the possible relationship between intestinal flora, nutrition preparation, and hormone axis, which lays a foundation for follow-up research, but its mechanism remains to be explored.

## Data availability statement

The data presented in this study are deposited in the NCBI Sequence Read Archive (SRA) under the Bio project number: PRJNA899648.

## Ethics statement

This animal study was approved by the Ethics Committee of Institute of Environmental and Operational Medicine.

## Author contributions

LJ, CG, DY, and WG conceived, designed, and wrote the manuscript. LJ, XB, RY, and WD executed the laboratory work and evaluated the data. LJ, XB, XL, CJ, and DY pre-processed the sequence data. All authors have contributed significantly to this work and approved the final version of the manuscript.

## References

[B1] Adlercreutz H. Pulkkinen M. Hämäläinen E. Korpela J. (1984). Studies on the role of intestinal bacteria in metabolism of synthetic and natural steroid hormones. *J. Steroid. Biochem.* 20 217–229. 10.1016/0022-4731(84)90208-5 6231418

[B2] Atarashi K. Tanoue T. Oshima K. Suda W. Nagano Y. Nishikawa H. (2013). T_reg_ induction by a rationally selected mixture of Clostridia strains from the human microbiota. *Nature* 500 232–236. 10.1038/nature12331 23842501

[B3] Bae J. Park K. Kim Y.-M. (2022). Commensal microbiota and cancer immunotherapy: Harnessing commensal bacteria for cancer therapy. *Immune Netw.* 22 e3. 10.4110/in.2022.22.e3 35291651 PMC8901697

[B4] Cao Y. Shen J. Ran Z. H. (2014). Association between Faecalibacterium prausnitzii reduction and inflammatory bowel disease: A meta-analysis and systematic review of the literature. *Gastroent. Res. Pract.* 2014:872725. 10.1155/2014/872725 24799893 PMC3985188

[B5] Chen K. (2020). *Effects of fermented Danggui Buxue decoction on growth performance and intestinal microflora diversity in broilers.* Master’s thesis. Beijing: Chinese Academy of Agricultural Sciences.

[B6] Chevalier C. Stojanović O. Colin D. J. Suarez-Zamorano N. Tarallo V. Veyrat-Durebex C. (2015). Gut microbiota orchestrates energy homeostasis during cold. *Cell* 163 1360–1374. 10.1016/j.cell.2015.11.004 26638070

[B7] Daher R. Yazbeck T. Jaoude J. B. Abboud B. (2009). Consequences of dysthyroidism on the digestive tract and viscera. *World J. Gastroentero.* 15:2834. 10.3748/wjg.15.2834 19533804 PMC2699000

[B8] Duan K. Gao X. Geng T. Cao L. Xiao W. Wang Z. (2020). Effects of guizhi fuling capsules and its main components on intestinal flora of primary dysmenorrhea model rats. *China Pharm.* 12, 1320–1326.

[B9] Einerhand A. W. Renes I. B. Makkink M. K. van der Sluis M. Büller H. A. Dekker J. (2002). Role of mucins in inflammatory bowel disease: Important lessons from experimental models. *Eur. J. Gastroen. Hepat.* 14 757–765. 10.1097/00042737-200207000-00008 12169985

[B10] Eom J. S. Lee S. Y. Choi H. S. (2014). Bacillus subtilis HJ18-4 from traditional fermented soybean food inhibits *Bacillus cereus* growth and toxin-related genes. *J. Food Sci.* 79 M2279–M2287. 10.1111/1750-3841.12569 25359543

[B11] Fröhlich E. Wahl R. (2019). Microbiota and thyroid interaction in health and disease. *Trends Endocrin. Met.* 30 479–490. 10.1016/j.tem.2019.05.008 31257166

[B12] Gong X. Li X. Bo A. Shi R.-Y. Li Q.-Y. Lei L.-J. (2020). The interactions between gut microbiota and bioactive ingredients of traditional Chinese medicines: A review. *Pharmacol. Res.* 157:104824. 10.1016/j.phrs.2020.104824 32344049

[B13] Grech-Mora I. Fardeau M.-L. Patel B. Ollivier B. Rimbault A. Prensier G. (1996). Isolation and characterization of *Sporobacter termitidis* gen. nov., sp. nov., from the digestive tract of the wood-feeding termite *Nasutitermes lujae*. *Int. J. Syst. Evol. Microbiol.* 46 512–518. 10.1099/00207713-46-2-512

[B14] Gresse R. Chaucheyras-Durand F. Fleury M. A. Van de Wiele T. Forano E. Blanquet-Diot S. (2017). Gut microbiota dysbiosis in postweaning piglets: Understanding the keys to health. *Trends Microbiol.* 25 851–873. 10.1016/j.tim.2017.05.004 28602521

[B15] Guowen C. Daiqin Z. Rongguo D. Yongkang J. Shulan Z. Shaoqin Z. (2003). Effects of Chinese herbal medicine additives on intestinal microflora and production performance of weaned Pigs. *Chin. Vet. Sci. Technol.* 33 54–58.

[B16] Hasnain S. Z. Wang H. Ghia J. E. Haq N. Deng Y. Velcich A. (2010). Mucin gene deficiency in mice impairs host resistance to an enteric parasitic infection. *Gastroenterology* 138 1763–1771. 10.1053/j.gastro.2010.01.045 20138044 PMC3466424

[B17] Homma H. Hoy E. Xu D. Z. Lu Q. Feinman R. Deitch E. A. (2005). The female intestine is more resistant than the male intestine to gut injury and inflammation when subjected to conditions associated with shock states. *Am. J. Physiol.* 288 G466–G472. 10.1152/ajpgi.00036.2004 15499084

[B18] Ishaq H. M. Mohammad I. S. Guo H. Shahzad M. Hou Y. J. Ma C. (2017). Molecular estimation of alteration in intestinal microbial composition in Hashimoto’s thyroiditis patients. *Biomed. Pharmacother.* 95 865–874. 10.1016/j.biopha.2017.08.101 28903182

[B19] Iszatt N. Janssen S. Lenters V. Dahl C. Stigum H. Knight R. (2019). Environmental toxicants in breast milk of Norwegian mothers and gut bacteria composition and metabolites in their infants at 1 month. *Microbiome* 7 1–14. 10.1097/01.EE9.0000607740.61316.16 30813950 PMC6393990

[B20] Ježová D. Juránková E. Mosnárová A. Kriška M. (1996). Neuroendocrine response during stress with relation to gender differences. *Acta Neurobiol. Exp.* 56 779–785. 10.55782/ane-1996-1183 8917906

[B21] Jiang Z. Cao L.-X. Liu B. Chen Q.-C. Shang W.-F. Zhou L. (2017). Effects of Chinese herbal medicine Xiangbin prescription on gastrointestinal motility. *World J. Gastroenterol.* 23:2987. 10.3748/wjg.v23.i16.2987 28522917 PMC5413794

[B22] Ju T. Kong J. Y. Stothard P. Willing B. P. (2019). Defining the role of *Parasutterella*, a previously uncharacterized member of the core gut microbiota. *ISME J.* 13 1520–1534. 10.1038/s41396-019-0364-5 30742017 PMC6776049

[B23] Kaliannan K. Robertson R. C. Murphy K. Stanton C. Kang C. Wang B. (2018). Estrogen-mediated gut microbiome alterations influence sexual dimorphism in metabolic syndrome in mice. *Microbiome* 6:205. 10.1186/s40168-018-0587-0 30424806 PMC6234624

[B24] Kawakami S. Ito R. Maruki-Uchida H. Kamei A. Yasuoka A. Toyoda T. (2020). Intake of a mixture of sake cake and rice malt increases mucin levels and changes in intestinal microbiota in mice. *Nutrients* 12:449. 10.3390/nu12020449 32053963 PMC7071214

[B25] Kinsey-Jones J. Li X. Knox A. Wilkinson E. Zhu X. Chaudhary A. (2009). Down-regulation of hypothalamic kisspeptin and its receptor, Kiss1r, mRNA expression is associated with stress-induced suppression of luteinising hormone secretion in the female rat. *J. Neuroendocrinol.* 21 20–29. 10.1111/j.1365-2826.2008.01807.x 19094090

[B26] Lee C. Hong S. N. Paik N. Y. Kim T. J. Kim E. R. Chang D. K. (2019). CD1d modulates colonic inflammation in NOD2^–/–^ mice by altering the intestinal microbial composition comprising *Acetatifactor muris*. *J Crohns Colitis* 13 1081–1091. 10.1093/ecco-jcc/jjz025 31094420

[B27] Lee H.-C. Yu S.-C. Lo Y.-C. Lin I.-H. Tung T.-H. Huang S.-Y. (2019). A high linoleic acid diet exacerbates metabolic responses and gut microbiota dysbiosis in obese rats with diabetes mellitus. *Food Funct.* 10 786–798. 10.1039/c8fo02423e 30672576

[B28] Lerner A. Jeremias P. Matthias T. (2017). Gut-thyroid axis and celiac disease. *Endocr. Connect.* 6 R52–R58. 10.1530/EC-17-0021 28381563 PMC5435852

[B29] Li T. Zhuang S. Wang Y. Wang Y. Wang W. Zhang H. (2016). Flavonoid profiling of a traditional Chinese medicine formula of Huangqin Tang using high performance liquid chromatography. *Acta Pharm. Sin. B* 6 148–157. 10.1016/j.apsb.2016.01.001 27006899 PMC4788706

[B30] Li W. Changsheng C. Jiangfang F. Bin G. Nanyan Z. Xiaomiao L. (2010). Effects of sex steroid hormones, thyroid hormone levels, and insulin regulation on thyrotoxic periodic paralysis in Chinese men. *Endocrine* 38 386–390. 10.1007/s12020-010-9396-3 20972724 PMC5454485

[B31] Li Y. Zheng D. Shen D. Zhang X. Zhao X. Liao H. (2020). Protective effects of two safflower derived compounds, kaempferol and hydroxysafflor yellow A, on hyperglycaemic stress-induced podocyte apoptosis via modulating of macrophage M1/M2 polarization. *J Immunol. Res.* 2020:2462039. 10.1155/2020/2462039 33102606 PMC7569436

[B32] Liang Z. Di N. Li L. Yang D. (2021). Gut microbiota alterations reveal potential gut–brain axis changes in polycystic ovary syndrome. *J. Endocrinol. Invest.* 44 1727–1737. 10.1007/s40618-020-01481-5 33387350

[B33] Liu Q.-l. (2005). Clinical application of peach seed compatibility. *Guiding J. Tcm* 11 65–66. 10.3969/j.issn.1672-951X.2005.10.034

[B34] Liu S. Zhang Y. Zhang M. Sun Y. Wei A. (2013). Research progress on producing mechanism and physiological functions of intestinal short chain fatty acids. *Guangdong Agric. Sci.* 11 99–103.

[B35] Lixia G. Weiwei P. Meiying J. Meiling W. Chunli P. (2019). Exploration about the clinical application and dosage of Angelica. *Jilin J. Tradit. Chin. Med.* 39 1013–1016,1020. 10.13463/j.cnki.jlzyy.2019.08.010

[B36] Liye Z. Chengwang T. Suxiang L. Changqing C. (2019). Prediction and Analysis of Chemical constituents, Pharmacological Action and quality Marker (Q-marker) of Guizhi Liling prescription. *Chin. Herb. Med.* 50 265–272.

[B37] Lozupone C. A. Stombaugh J. I. Gordon J. I. Jansson J. K. Knight R. (2012). Diversity, stability and resilience of the human gut microbiota. *Nature* 489 220–230. 10.1038/NATURE11550 22972295 PMC3577372

[B38] Meng S. Badrinarain J. Sibley E. Fang R. Hodin R. (2001). Thyroid hormone and the d-type cyclins interact in regulating enterocyte gene transcription. *J. Gastrointest. Surg.* 5 49–55. 10.1016/s1091-255x(01)80013-5 11309648

[B39] Nazhand A. Lucarini M. Durazzo A. Zaccardelli M. Cristarella S. Souto S. B. (2020). Hawthorn (*Crataegus* spp.): An updated overview on its beneficial properties. *Forests* 11:564.

[B40] Opyd P. M. Jurgoński A. Juśkiewicz J. Milala J. Zduńczyk Z. Król B. (2017). Nutritional and health-related effects of a diet containing apple seed meal in rats: The case of amygdalin. *Nutrients* 9:1091. 10.3390/nu9101091 28974035 PMC5691708

[B41] Pääkkönen T. Leppäluoto J. (2002). Cold exposure and hormonal secretion: A review. *Int. J. Circumpolar Health* 61 265–276. 10.3402/ijch.v61i3.17474 12369117

[B42] Qin J. Zhou J. Fan C. Zhao N. Liu Y. Wang S. (2017). Increased circulating Th17 but decreased CD4^+^ Foxp3^+^ Treg and CD19^+^ CD1dhiCD5^+^ Breg subsets in new-onset Graves’ disease. *Biomed. Res. Int.* 2017:8431838. 10.1155/2017/8431838 29259988 PMC5702927

[B43] Qu Y. Li X. Xu F. Zhao S. Wu X. Wang Y. (2021). Kaempferol alleviates murine experimental colitis by restoring gut microbiota and inhibiting the LPS-TLR4-NF-κB Axis. *Front. Immunol.* 12:679897. 10.3389/fimmu.2021.679897 34367139 PMC8339999

[B44] Sakamoto M. Takagaki A. Matsumoto K. Kato Y. Goto K. Benno Y. (2009). *Butyricimonas synergistica* gen. nov., sp. nov. and *Butyricimonas virosa* sp. nov., butyric acid-producing bacteria in the family ‘Porphyromonadaceae’isolated from rat faeces. *Int. J. Syst. Evol. Microbiol.* 59 1748–1753. 10.1099/ijs.0.007674-0 19542124

[B45] Selye H. (1936). A syndrome produced by diverse nocuous agents. *Nature* 138:32. 10.1038/138032a0 9722327

[B46] Seo B. Jeon K. Moon S. Lee K. Kim W.-K. Jeong H. (2020). *Roseburia* spp. abundance associates with alcohol consumption in humans and its administration ameliorates alcoholic fatty liver in mice. *Cell Host Microbe* 27 25–40.e26. 10.1016/j.chom.2019.11.001 31866426

[B47] Shi D. Lv L. Fang D. Wu W. Hu C. Xu L. (2017). Administration of *Lactobacillus salivarius* LI01 or *Pediococcus pentosaceus* LI05 prevents CCl4-induced liver cirrhosis by protecting the intestinal barrier in rats. *Sci. Rep.* 7 1–13. 10.1038/s41598-017-07091-1 28761060 PMC5537250

[B48] Shi T. Bian X. Yao Z. Wang Y. Gao W. Guo C. (2020). Quercetin improves gut dysbiosis in antibiotic-treated mice. *Food Funct.* 11 8003–8013. 10.1039/d0fo01439g 32845255

[B49] Sicard J.-F. Le Bihan G. Vogeleer P. Jacques M. Harel J. (2017). Interactions of intestinal bacteria with components of the intestinal mucus. *Front Cell Infect. Microbiol.* 7:387. 10.3389/fcimb.2017.00387 28929087 PMC5591952

[B50] Strandwitz P. Kim K. H. Terekhova D. Liu J. K. Sharma A. Levering J. (2019). GABA-modulating bacteria of the human gut microbiota. *Nat. Microbiol.* 4 396–403. 10.1038/s41564-018-0307-3 30531975 PMC6384127

[B51] Tang C. W. Zhu M. Feng W. M. Bao Y. Zheng Y. Y. (2016). Chinese herbal medicine, Jianpi Ligan decoction, improves prognosis of unresectable hepatocellular carcinoma after transarterial chemoembolization: A retrospective study. *Drug Des. Devel. Ther.* 10:2461. 10.2147/DDDT.S113295 27536066 PMC4977068

[B52] Tian B. Geng Y. Wang P. Cai M. Neng J. Hu J. (2022). Ferulic acid improves intestinal barrier function through altering gut microbiota composition in high-fat diet-induced mice. *Eur. J. Nutr.* 61 3767–3783. 10.1007/s00394-022-02927-7 35732902

[B53] Ubeda C. Bucci V. Caballero S. Djukovic A. Toussaint N. C. Equinda M. (2013). Intestinal microbiota containing *Barnesiella* species cures vancomycin-resistant *Enterococcus faecium* colonization. *Infect. Immun.* 81 965–973. 10.1128/IAI.01197-12 23319552 PMC3584866

[B54] Vanhoutvin S. A. Troost F. J. Hamer H. M. Lindsey P. J. Koek G. H. Jonkers D. M. (2009). Butyrate-induced transcriptional changes in human colonic mucosa. *PLoS One* 4:e6759. 10.1371/journal.pone.0006759 19707587 PMC2727000

[B55] Wang D. Cheng X. Fang H. Ren Y. Li X. Ren W. (2020). Effect of cold stress on ovarian & uterine microcirculation in rats and the role of endothelin system. *BioMed Central* 18:29. 10.1186/s12958-020-00584-1 32290862 PMC7155299

[B56] Wang Q. Zhou L. Guo Y. Liu G. Cheng J. Yu H. (2013). Differentiation of human adipose-derived stem cells into neuron-like cells by Radix Angelicae Sinensis. *Neural Regen. Res.* 8:3353. 10.3969/j.issn.1673-5374.2013.35.010 25206657 PMC4145944

[B57] Wostmann B. S. (2020). *Germfree and gnotobiotic animal models: Background and applications.* Boca Raton, FL: CRC Press.

[B58] Xinhao Z. Dongke X. Hao Z. Pei X. Xiaoyong W. (2022). Protective effect of amygdalin on necrotizing enterocolitis in neonatal rats. *Chin. J. Comp. Med.* 32 75–81.

[B59] Xu H.-y. Wang Y.-l. Wang D.-f. Li H.-x. Yang W.-p. (2017). Effect of Huangqin Tang on the gut microbiota in rats with ulcerative colitis model determined by high-throughput sequencing. *Acta Pharm. Sin.* 12, 1673–1682.

[B60] Xu T. Li X. Yang L. Zhang Y. Zhang L. Guo Z. (2018). Impact of cold exposure on the reproductive function in female rats. *Biomed. Res. Int.* 2018:3674906. 10.1155/2018/3674906 30596088 PMC6282150

[B61] Yang J. A. Song C. I. Hughes J. K. Kreisman M. J. Parra R. A. Haisenleder D. J. (2017). Acute psychosocial stress inhibits LH pulsatility and Kiss1 neuronal activation in female mice. *Endocrinology* 158 3716–3723. 10.1210/en.2017-00301 28973125 PMC5695836

[B62] Yusufu I. Ding K. Smith K. Wankhade U. D. Sahay B. Patterson G. T. (2021). A tryptophan-deficient diet induces gut microbiota dysbiosis and increases systemic inflammation in aged mice. *Int. J Mol. Sci.* 22:5005. 10.3390/ijms22095005 34066870 PMC8125914

[B63] Zhang X. Cui X. Jin X. Han F. Wang J. Yang X. (2020). Preventive role of salsalate in diabetes is associated with reducing intestinal inflammation through improvement of gut dysbiosis in ZDF rats. *Front. Pharmacol.* 11:300. 10.3389/fphar.2020.00300 32265702 PMC7096544

[B64] Zhang Z. W. Lv Z. H. Li J. L. Li S. Xu S. W. Wang X. L. (2011). Effects of cold stress on nitric oxide in duodenum of chicks. *Poult. Sci.* 90 1555–1561. 10.3382/ps.2010-01333 21673172

[B65] Zhao F. Feng J. Li J. Zhao L. Liu Y. Chen H. (2018). Alterations of the gut microbiota in Hashimoto’s thyroiditis patients. *Thyroid* 28 175–186. 10.1089/thy.2017.0395 29320965

[B66] Zhao G. Nyman M. (2006). Rapid determination of short-chain fatty acids in colonic contents and faeces of humans and rats by acidified water-extraction and direct-injection gas chromatography. *Biomed. Chromatogr.* 20 674–682. 10.1002/bmc.580 16206138

[B67] Ziętak M. Kovatcheva-Datchary P. Markiewicz L. H. Ståhlman M. Kozak L. P. Bäckhed F. (2016). Altered microbiota contributes to reduced diet-induced obesity upon cold exposure. *Cell Metab.* 23 1216–1223. 10.1016/j.cmet.2016.05.001 27304513 PMC4911343

[B68] Zivkovic A. M. German J. B. Lebrilla C. B. Mills D. A. (2011). Human milk glycobiome and its impact on the infant gastrointestinal microbiota. *Proc. Natl. Acad. Sci.* 108(Suppl. 1) 4653–4658. 10.1073/pnas.1000083107 20679197 PMC3063602

